# Первичный гипотиреоз и постменопауза как причины отсроченной диагностики пангипопитуитаризма у пациентки с гормонально-неактивной аденомой гипофиза

**DOI:** 10.14341/probl13128

**Published:** 2022-07-05

**Authors:** Е. Г. Рыжкова, Д. О. Ладыгина

**Affiliations:** Центральная клиническая больница с поликлиникой Управления делами Президента Российской Федерации; Центральная клиническая больница с поликлиникой Управления делами Президента Российской Федерации; Клиника Фомина

**Keywords:** клинический случай, гипопитуитаризм, гормонально-неактивное образование гипофиза, центральный гипотиреоз, первичный гипотиреоз, постменопауза

## Abstract

Гипопитуитаризм — состояние полного или частичного дефицита гормонов гипофиза, включающее надпочечниковую недостаточность, гипотиреоз, гипогонадизм, недостаточность гормона роста и, реже, несахарный диабет.

В статье описан клинический случай гипопитуитаризма вследствие образования гипофиза у женщины в постменопаузе. Сложности диагностики гипопитуитаризма были обусловлены наличием в анамнезе первичного гипотиреоза, в результате чего первый из выявленных компонентов пангипопитуитаризма (центральный гипотиреоз) длительное время расценивался как лабораторный признак медикаментозного тиреотоксикоза. Неспецифический характер клинических симптомов, а также относительно редкое сочетание эндокринных заболеваний стали причиной длительного обследования и отсроченной диагностики образования хиазмально-селлярной области.

Вопрос о том, является ли развитие гипопитуитаризма у пациентов с гормонально-неактивными образованиями ­гипоталамо-гипофизарной области показанием к нейрохирургическому вмешательству, остается спорным. Принятие решения проводится с учетом особенностей течения заболевания у конкретного пациента. В приведенном клиническом случае была выбрана консервативная тактика с проведением заместительной терапии недостаточности глюкокортикоидных и тиреоидных гормонов.

## Abstract (en)

Hypopituitarism is a state of complete or partial deficiency of pituitary hormones, including adrenal insufficiency, hypothyroidism, hypogonadism, growth hormone deficiency, and, rarely, diabetes insipidus.

The article describes a clinical case of hypopituitarism due to a pituitary tumor in a postmenopausal woman. Difficulties in diagnosing hypopituitarism were due to a history of primary hypothyroidism. The first identified component of panhypopituitarism in the patient, (central hypothyroidism) had previously been seen as laboratory indications of medication-induced hyperthyroidism.

The non-specific nature of the clinical symptoms, as well as a relatively rare combination of endocrine diseases, led to a long examination period and delayed diagnosis of the pituitary tumor.

Whether the development of hypopituitarism in a patient with a nonfunctional pituitary tumor is an indication for transsphenoidal pituitary surgery remains a controversial issue. The decision for surgery is made taking into account the characteristics of the course of the disease in a particular patient. In this clinical case, a conservative tactic was chosen with hormone replacement therapy for glucocorticoid and thyroid deficiency.

## ОБОСНОВАНИЕ

Гипопитуитаризм возникает в результате полного или частичного дефицита гормонов гипофиза и включает надпочечниковую недостаточность, гипотиреоз, гипогонадизм, недостаточность гормона роста и, реже, несахарный диабет. Развитие заболевания обусловлено нарушением либо непосредственно секреторной функции гипофиза, либо стимулирующего воздействия гипоталамуса, что проявляется недостаточностью функции соответствующих органов периферической эндокринной системы [1].

Аденома гипофиза, хирургические вмешательства и лучевая терапия в области гипоталамо-гипофизарной системы являются наиболее частыми причинами гипопитуитаризма у взрослых. Профиль недостаточности гормонов гипофиза зависит от места повреждения в пределах гипоталамо-гипофизарной оси и характера патологического процесса [2]. В Европе распространенность дефицита гормонов передней доли гипофиза составляет 45,5 случая на 100 000 человек, ежегодная заболеваемость — от 4 до 21 случаев на 100 000 населения [3].

Диагностика гипопитуитаризма основана на оценке клинической картины и данных лабораторных методов исследования. Зачастую пациенты с нарушением секреции гормонов аденогипофиза имеют неспецифические и/или стертые симптомы. Это затрудняет выделение патогномоничных признаков дефицита гормонов и приводит к несвоевременной постановке диагноза [4]. Особенно сложно заподозрить гипопитуитаризм у пациентов пожилого и старческого возраста, а также при наличии первичной недостаточности периферических эндокринных желез.

В данной статье описан клинический случай пангипопитуитаризма у женщины в постменопаузе с длительным анамнезом первичного гипотиреоза. Описываемое клиническое наблюдение представляет интерес в связи со сложностью своевременной диагностики гипопитуитаризма в пожилом и старческом возрасте, особенно при наличии первичной недостаточности одного из гипофиз-зависимых органов вследствие аутоиммунного заболевания.

## ОПИСАНИЕ СЛУЧАЯ

Пациентка А., 69 лет, находилась на лечении в клиническом санатории Московской области в июле 2021 г. Направлена к врачу-эндокринологу с жалобами на общую слабость; эпизодическую головную боль, купирующуюся приемом ненаркотических анальгетиков; сниженный аппетит; острые переживания любых жизненных ситуаций; пробуждения ночью, сопровождающиеся ощущением нехватки воздуха, жаром в теле, чувством страха, позывами на мочеиспускание и повышением артериального давления (АД) до 160/100 мм рт.ст., несмотря на прием антигипертензивных препаратов.

Со слов пациентки, в 19 лет впервые выявлено повышение уровня тиреотропного гормона (ТТГ) более 10 мкМЕ/мл, диагностирован первичный гипотиреоз, назначена заместительная терапия левотироксином натрия 50 мкг в сутки. При регулярном определении уровень ТТГ находился в референсном интервале, доза левотироксина натрия не корректировалась.

По данным предоставленной медицинской документации от мая 2013 г., ТТГ 4,8 мкМЕ/мл (0,46–4,68). При ультразвуковом исследовании (УЗИ) щитовидной железы — объем 11 мл, эхографические признаки диффузных изменений паренхимы по типу тиреоидита. Доза левотироксина натрия была увеличена до 75 мкг в сутки.

Вышеописанные жалобы стали беспокоить с 2019 г. после перенесенной стрессовой ситуации. Тогда же была проведена магнитно-резонансная томография (МРТ) головного мозга, по результатам которой структурных изменений гипофиза не выявлено.

При гормональном исследовании в январе 2019 г. уровень ТТГ составил 5 мкМЕ/мл (0,46–4,68), антитела к тиреопероксидазе (АТ к ТПО) 289 МЕ/мл (менее 5,6 МЕ/мл). По месту жительства доза левотироксина натрия была увеличена до 88 мкг в сутки. В связи с ухудшением общего самочувствия с апреля 2021 г. проводились неоднократное исследование функции щитовидной железы (табл. 1) и коррекция заместительной терапии. Предпринималась попытка отмены левотироксина с дальнейшим возобновлением приема препарата по рекомендации эндокринологов различных лечебных учреждений.

**Table:** Таблица 1. Динамика лабораторных показателейTable 1. Dynamics of laboratory parameters Сокращения: св. Т3 — свободный трийодтиронин.

Показатели	2013	2019	2021	Референсныйинтервал
		Январь	Апрель	Май	Июнь	Июль	Август	Сентябрь	Ноябрь
Гормональное исследование
ТТГ, мкМЕ/мл	4,8	5	0,015	0,015	0,01	0,2				0,46–4,68
Св.Т4, пмоль/л			13,1	5,93	7,69	5,96	14,7	16,9	20,20	10–28,2
Св.Т3, пмоль/л										
АКТГ, пг/мл						18,3		7,62		0–46
Кортизол крови, нмоль/л						182		81,2; 82,3		138–690
ПРЛ, мкМЕ/мл						1479		64,7		102–496
ЛГ, мМЕ/мл						0,48				7,7–59
ФСГ, мМЕ/мл						2,33				14,8–25,8
ИФР1, нг/мл						62,9				37–219
ПТГ, пг/мл						34,97				15–65
Биохимическое исследование
Натрий, ммоль/л						139		140,08	141,9	136–145
Калий, ммоль/л						4,4		4,42	4,5	3,5–5,1
Глюкоза, ммоль/л						4,96		4,97	4,6	3,9–6,1
Проводимое лечение, доза
Левотироксин натрия, мкг/сут	75	88	отмена	62,5	25	75	100	100	100	
Гидрокортизон, мг/сут								15	15	

При осмотре на момент госпитализации в санаторий в июле 2021 г. клинические признаки акромегалии, гиперкортицизма отсутствовали. Подкожножировая клетчатка развита умеренно, распределена равномерно. Рост 174 см, вес 74 кг (ИМТ=24,3 кг/м2). Оволосение по женскому типу, вторичные половые признаки развиты правильно. Щитовидная железа пальпаторно не увеличена, плотная, неоднородная. В остальном по органам и системам без особенностей.

По данным гинекологического анамнеза, у пациентки было 5 беременностей, из них 3 завершились срочными родами и 2 — медицинскими абортами. На момент обращения постменопауза длительностью 14 лет.

На момент поступления, помимо левотироксина натрия в дозе 25 мкг в сутки, пациентка принимала селективный ингибитор обратного захвата серотонина пароксетин, ситуационно — анксиолитик бензодиазепинового ряда и гидроксизина гидрохлорид, а также комбинированную антигипертензивную терапию.

Результаты гормонального анализа крови от июля 2021 г. свидетельствовали о наличии центрального гипотиреоза (табл. 1). При биохимическом исследовании — признаки дислипидемии: повышение уровня общего холестерина до 7,4 ммоль/л, триглицеридов до 2,39 ммоль/л; повышение уровня печеночных трансаминаз до 1,5 норм. Результаты общеклинических анализов крови и мочи без клинически значимых изменений.

В связи с повторяющимися эпизодами панических атак в ночное время пациентка консультирована психиатром. Диагностировано генерализованное тревожное расстройство, проведена коррекция терапии.

С целью уточнения этиологии центрального гипотиреоза проведена МРТ гипофиза: в аксиальной и коронарной плоскостях в Т2-, Т2d-f- и Т1-режимах, интраселлярно, с распространением супраселлярно определяется объемное образование неправильной округлой формы с четкими, неровными контурами, неоднородной структуры, размерами 1,4×1,1×1,2 см, пониженного МР-сигнала в режиме Т1, повышенного в Т2, Т2df. Отмечаются признаки объемного воздействия: сглажено супраселлярное цистернальное пространство, хиазма оттеснена кверху, воронка гипофиза оттеснена вправо. После введения контрастного вещества отмечается слабое неоднородное накопление последнего небольшой зоной, размером до 4 мм (рис. 1 в, г).

**Figure:**
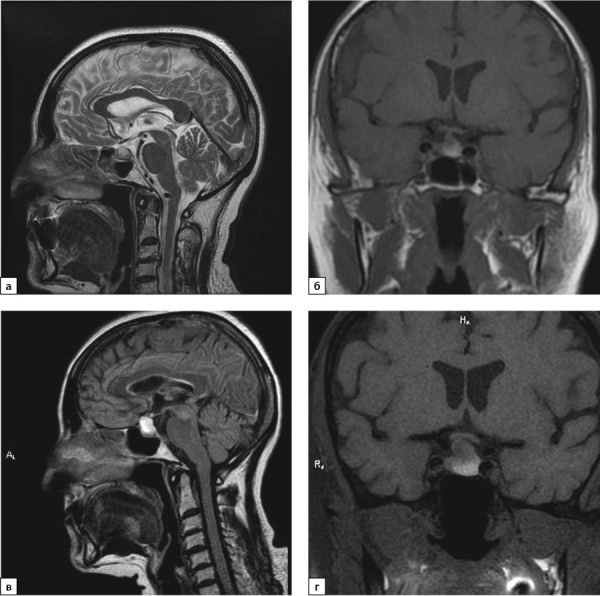
Рисунок 1. МРТ от 2019 г.: а) Т2-ВИ, сагиттальный срез; б) Т1-ВИ, корональный срез. МРТ от 2021 г.: в) Т2 FLAIR-ВИ, сагиттальный срез; г) Т1-ВИ, корональный срез.Примечание. ВИ — взвешенное изображение.Figure 1. MRI from 2019: a) T2-WI, sagittal section; b) T1-WI, coronal section. MRI from 2021: c) T2 FLAIR-VI, sagittal section; d) T1-WI, coronal section

При мультиспиральной компьютерной томографии надпочечников структурных изменений не выявлено.

При расширенном гормональном исследовании определялись гиперпролактинемия до 5 норм, снижение уровня лютеинизирующего гормона (ЛГ) и фолликулостимулирующего гормона (ФСГ) (табл. 1). Адренокортикотропный гормон (АКТГ), базальный кортизол сыворотки, инсулиноподобный фактор роста 1 (ИФР1) и паратиреоидный гормон (ПТГ) находились в пределах референсного интервала (табл. 1). Исключение феномена макропролактинемии не проводилось. Повышение уровня пролактина (ПРЛ) было расценено как вторичная гиперпролактинемия вследствие сдавления ножки гипофиза и нарушения транспорта дофамина.

23.07.2021 г. пациентка консультирована в НМИЦ нейрохирургии имени академика Н.Н. Бурденко. По данным осмотра нейроофтальмолога в ходе компьютерной периметрии сужений полей зрения не выявлено. Острота зрения: OD 0,7 D, OS до 0,7 D. По заключению нейрохирурга абсолютных показаний к проведению хирургического вмешательства на момент осмотра нет, рекомендовано динамическое наблюдение (проведение МРТ гипофиза и компьютерной периметрии через 6 мес).

На основании клинических, лабораторных и инструментальных данных был сформулирован следующий диагноз: эндосупраселлярная опухоль гипофиза (гормонально-неактивная). Смешанный гипогонадизм (первичный, вторичный). Гипотиреоз сочетанного генеза (первичный, вторичный). Вторичная гиперпролактинемия.

Принимая во внимание отсутствие нарушений зрительной функции и желание пациентки, рекомендована консервативная тактика: назначение агониста дофаминовых рецепторов (каберголин) 0,25 мг 2 раза в неделю с постепенным увеличением дозы до антипролиферативной (3,5 мг в неделю) под контролем переносимости; заместительная терапия левотироксином натрия 75 мкг в сутки.

В ходе пребывания пациентки в санатории отмечены эпизоды снижения артериального давления до 100/70 мм рт. ст., были отменены антигипертензивные препараты. Учитывая генерализованное тревожное расстройство, рекомендовано продолжить прием антидепрессанта эсциталопрама в дозе 10 мг в стуки и анксиолитика бензодиазепинового ряда ситуационно.

В дальнейшем пациентка наблюдалась амбулаторно по месту жительства. По результатам гормонального исследования от августа 2021 г. увеличены дозы левотироксина натрия и каберголина (табл. 1). Отмечались колебания АД от 90/60 мм рт. cт.до 130/80 мм рт. ст.

С середины сентября 2021 г. развилась тенденция к снижению АД до 80/50 мм рт.ст в дневные часы, присоединилось ощущение скованности в суставах, наросла общая слабость, неспособность выполнения обычных дел по дому. По результатам исследования уровня АКТГ и кортизола сыворотки двукратно подтверждена надпочечниковая недостаточность (НН). Уровень свободного тироксина (св.Т4), глюкозы и электролитов находился в пределах референсного интервала (табл. 1). Рекомендован прием гидрокортизона внутрь 10 мг утром и 5 мг в 14.00, на фоне чего отмечены улучшение самочувствия, стабилизация АД в пределах 110/70–130/80 мм рт.ст.

В ноябре 2021 г. пациентка по собственному желанию в одной из клиник г. Москвы выполнила МРТ головного мозга. Сохраняется МР-картина эндосупраселлярного объемного образования размерами 18×13×14 мм, в дифференциальный диагноз следует включить макроаденому гипофиза и краниофарингиому. При сравнении с представленными снимками МРТ от 11.11.2019 г. отмечается увеличение размеров образования, от 27.07.2021 г. — динамических изменений нет. Таким образом, ревизия МРТ исследования от 2019 г. свидетельствовала об имевшемся, но не описанном ранее образовании гипофиза меньших размеров (рис. 1а, б).

Тогда же при повторном осмотре офтальмологом с проведением компьютерной периметрии нейроофтальмологическая симптоматика отсутствовала.

При лабораторном обследовании: уровень св.Т4 находился в верхнем квартиле референсного диапазона, глюкоза и электролиты — в пределах референсного интервала (табл. 1). Консультирована нейрохирургом: операция может быть проведена в связи с ростом образования по сравнению с 2019 г., прилежанием его к зрительному перекресту и развитием гипопитуитаризма. Учитывая отказ пациентки от хирургического вмешательства и отсутствие нарушений полей зрения, рекомендовано продолжить прием левотироксина натрия и гидрокортизона в прежних дозах, каберголина с постепенным увеличением дозы до 3,5 мг в неделю; контроль МРТ головного мозга и исследование полей зрения через 6 месяцев.

На фоне коррекции терапии анксиолитиками и антидепрессантами исчезли ночные панические атаки. Достигнуты стабильные цифры АД, пациентка вернулась к прежнему уровню физической активности.

## ОБСУЖДЕНИЕ

Представленный клинический случай иллюстрирует сложность диагностики гормонально-неактивных образований гипофиза в постменопаузе. Особенностью данного случая стало развитие образования гипоталамо-гипофизарной области упациентки с первичным гипотиреозом в постменопаузе, что отсрочило своевременную постановку диагноза.

Среди опухолей хиазмально-селлярной области причиной недостаточности тропных гормонов передней доли гипофиза чаще всего является гормонально неактивная аденома гипофиза [2]. Другими причинами гипопитуитаризма у взрослых могут быть повреждение гипоталамо-гипофизарной области вследствие травмы, облучения или хирургического вмешательства, развития инфекционных и аутоиммунных заболеваний [4].

При наличии опухоли гипофиза основными предполагаемыми патофизиологическими механизмами дефицита гормонов являются сжатие воротной системы в ножке гипофиза, прямое механическое давление или повреждение железы опухолевой массой, повышенное внутриселлярное давление и очаговый некроз вследствие длительного пережатия воротной вены [5].

Клиническая картина недостаточности тропных гормонов в сравнении с нарушением функции периферических эндокринных желез характеризуется более мягким течением и стертой симптоматикой.

В подавляющем большинстве случаев нарушение секреции тропных гормонов гипофиза происходит по классической модели: в первую очередь и наиболее часто уменьшается продукция соматотропного гормона, за которым следует дефицит гонадотропинов (ЛГ и ФСГ), далее ТТГ, АКТГ и ПРЛ [5]. Однако из данного правила могут быть исключения, обусловленные этиологией процесса в хиазмально-селлярной области [1].

Исходя из «классической» последовательности нарушения секреции гормонов гипофиза на фоне роста опухоли, у взрослых первые симптомы гипопитуитаризма обусловлены дефицитом гонадотропных гормонов, что включает нарушение менструальной функции у женщин, низкий уровень тестостерона у мужчин и бесплодие вне зависимости от пола.

Учитывая возраст пациентки, клинические проявления дефицита гонадотропинов отсутствовали. Наиболее частыми клиническими проявлениями образования гипофиза размером более 1 см в возрасте после 50 лет являются симптомы масс-эффекта, которых также не наблюдалось у данной пациентки [6].

Первичным лабораторным проявлением гипопитуитаризма в представленном случае было развитие центрального гипотиреоза (ЦГ). ЦГ характеризуется нарушением синтеза тиреоидных гормонов вследствие недостаточности стимулирующего действия ТТГ на неизмененную щитовидную железу. Причиной могут быть функциональные или структурные нарушения гипоталамуса (третичный гипотиреоз), следствием чего является нарушение секреции тиролиберина и/или гипофиза (вторичный гипотиреоз), с нарушением секреции ТТГ [7].

Клинические признаки ЦГ, как и первичного, весьма неспецифичны и не могут служить достоверным критерием постановки диагноза. Диагностическим критерием ЦГ является сочетание низкого уровня св.Т4 с низким или нормальным уровнем ТТГ [7][8].

В представленном клиническом примере на фоне приема различных доз левотироксина натрия отчетливо прослеживались типичные лабораторные признаки ЦГ. Примечательно, что на момент обследования пациентка длительное время принимала левотироксин натрия по поводу первичного гипотиреоза (ПГ) в исходе хронического аутоиммунного тиреоидита. Ситуация была расценена как медикаментозный тиреотоксикоз, в связи с чем левотироксин натрия неоднократно отменялся или снижалась его доза. Оценка только уровня ТТГ, что является общепринятой практикой при ПГ, также послужило одной из причин отсроченной диагностики ЦГ у пациентки [9][10].

Несоответствие динамики лабораторных показателей типичной картине, наблюдаемой при лечении ПГ, позволило заподозрить, а затем и подтвердить ЦГ. После этого контроль адекватности заместительной терапии проводился по уровню св.Т4. Целью проводимой заместительной терапии является значение св.Т4 в верхнем квартиле референсного диапазона [7–9].

Согласно ряду рекомендаций, перед назначением левотироксина натрия пациентам c ЦГ, имеющим образование гипофиза, рекомендовано исключение НН [1][8][9]. В случае, если сопутствующая центральная надпочечниковая недостаточность (ЦНН)не исключена, лечение гипотиреоза рекомендовано начинать только после эмпирического назначения глюкокортикостероидов во избежание развития острой НН. Это обусловлено тем, что гормоны щитовидной железы ускоряют метаболизм кортизола впечени и их назначение может спровоцировать развитие острой НН у пациентов с частично утраченной секрецией АКТГ [1].

Точкой «cut off», при которой диагноз ЦНН высоковероятен и проведение функциональных проб не показано, является уровень кортизола сыворотки крови в ранние утренние часы (06.00–08.00) менее 83 нмоль/л (3 г/дл) в сочетании с нормальным или низким уровнем АКТГ [1]. В описываемом примере пероральный гидрокортизон добавлен к лечению через 2 мес после выявления ЦГ в связи с клиническими и лабораторными признаками ЦНН. Уровень кортизола крови утром при двукратном определении составлял менее 83 нмоль/л, а уровень АКТГ находился в нижнем квартиле референсного интервала.

Учитывая склонность к более низким значениям АД, что потребовало отмены антигипертензивной терапии, низконормальный уровень кортизола (182 нмоль/л) и натрия (139 ммоль/л) в сыворотке на момент выявления ЦГ, можно предположить, что у пациентки уже была ЦНН. Таким образом, имелись показания к назначению гидрокортизона перед увеличением дозы левотироксина натрия. Следует отметить, что пациентка по своей инициативе неоднократно консультировалась у различных нейрохирургов и эндокринологов в специализированных медицинских учреждениях, в части из которых ей было рекомендовано прекратить прием гидрокортизона, несмотря на вышеописанные клинические и лабораторные признаки НН. Это подчеркивает важность вынесенной на обсуждение темы.

Большинство случаев пангипопитуитаризма, вызванных гормонально-неактивными образованиями хиазмально-селлярной области, характеризуется повышением уровня ПРЛ, что обычно обусловлено нарушением транспорта дофамина [1]. Несмотряна то что причинами гиперпролактинемии в описываемом случае могли явиться как гипотиреоз в стадии декомпенсации, так и прием антидепрессанта, нарушение транспорта дофамина является наиболее вероятной причиной. При этом уровень ПРЛ при первичном определении менее 2000 мЕд/л и его снижение после начала приема каберголина до 64,7 мкМЕ/мл (102–496) также подтверждают вторичный характер его повышения [11][12].

Вопрос о проведении хирургического лечения в случае развития гипопитуитаризма как единственного клинического проявления нефункционирующего образования хиазмально-селлярной области остается спорным [13]. По данным Huang W. et al. [14], частота восстановления секреции тропных гормонов у этой категории пациентов после транссфеноидального вмешательства широко варьирует.

Учитывая отсутствие гарантии восстановления секреторной функции передней доли гипофиза после хирургического лечения, гипопитуитаризм не может рассматриваться в качестве абсолютного показания к оперативному вмешательству [14]. При этом необходимость проведения адекватной заместительной терапии не зависит от выбранной тактики [13].

Имеются литературные данные о том, что нормальный или умеренно повышенный уровень ПРЛ до операции предполагает адекватный (сохраненный) гипофизарный резерв и прогнозирует более высокий шанс восстановления секреторной функции аденогипофиза [15].

Интимное прилегание образования к хиазме в представленном клиническом случае может рассматриваться в качестве относительного показания к оперативному лечению. Однако данная тактика чаще применяется у молодых пациентов в связи с большей вероятностью увеличения опухоли, в то время как у пациентов старше 65 лет выше риск послеоперационных осложнений [13][16][17].

Согласно рекомендациям Российского общества эндокринологов и международного эндокринологического общества, абсолютным показанием к хирургическому лечению у пациентов с инциденталомами гипофиза является наличие зрительных нарушений [13][16].

При наличии гормонально-неактивной макроаденомы гипофиза и отсутствии абсолютных показаний к нейрохирургическому лечению может быть рекомендован прием агонистов дофамина с антипролиферативной целью, с достижением дозы каберголина 3,5 мг в неделю [16]. Назначение агонистов дофамина пациентам с гормонально-неактивными образованиями гипофиза обосновано экспрессией дофаминовых рецепторов 2 типа в данном типе опухоли [18]. Несколько клинических исследований показало, что назначение бромокриптина в дозе от 15 до 60 мг в сутки при первичном лечении гормонально-неактивных образований гипофиза приводило к стабилизации размеров опухоли. У 28–67% пациентов, которые ранее перенесли хирургическое вмешательство, лечение каберголином приводило к уменьшению размеров опухоли более чем на 25% [19]. С учетом относительно больших размеров образования у данной пациентки, его прилегания к хиазме, было решено назначить каберголин в антипролиферативной дозе (3,5 мг в неделю) с последующим динамическим наблюдением под контролем переносимости.

## ЗАКЛЮЧЕНИЕ

В представленном клиническом случае образование гипофиза было диагностировано благодаря выявлению одного из компонентов гипопитуитаризма — ЦГ, несмотря на длительный анамнез ПГ и проведение обследования в период менопаузы.

Мы полагаем, что разбор данного клинического случая обратит внимание на проблему своевременной диагностики гормонально-неактивных образований гипофиза в пожилом и старческом возрасте как в общей массе, так и у пациентов с другими эндокринными заболеваниями.

## ДОПОЛНИТЕЛЬНАЯ ИНФОРМАЦИЯ

Источники финансирования. Работа выполнена по инициативе авторов без привлечения финансирования.

Конфликт интересов. Авторы декларируют отсутствие явных и потенциальных конфликтов интересов, связанных с содержанием настоящей статьи.

Участие авторов. Рыжкова Е.Г. — существенный вклад в концепцию и дизайн исследования, написание статьи; Ладыгина Д.О. — существенный вклад в концепцию и дизайн исследования, внесение в рукопись существенной правки с целью повышения научной ценности статьи;

Все авторы одобрили финальную версию статьи перед публикацией, выразили согласие нести ответственность за все аспекты работы, подразумевающую надлежащее изучение и решение вопросов, связанных с точностью или добросовестностью любой части работы.

Согласие пациента. Пациент добровольно подписал информированное согласие на публикацию персональной медицинской информации в обезличенной форме.
